# Flagellin-fused protein targeting M2e and HA2 induces potent humoral and T-cell responses and protects mice against various influenza viruses a subtypes

**DOI:** 10.1186/s12929-018-0433-5

**Published:** 2018-04-09

**Authors:** Liudmila A. Stepanova, Eugenia S. Mardanova, Marina A. Shuklina, Elena A. Blokhina, Roman Y. Kotlyarov, Marina V. Potapchuk, Anna A. Kovaleva, Inna G. Vidyaeva, Alexandr V. Korotkov, Elizaveta I. Eletskaya, Nikolai V. Ravin, Liudmila M. Tsybalova

**Affiliations:** 1 Research Institute of Influenza, Russian Ministry of Health, Prof. Popova str.15/17, 197376 St. Petersburg, Russia; 2 Institute of Bioengineering, Research Center of Biotechnology, Russian Academy of Sciences, Leninsky Ave. 33, building 2, 119071 Moscow, Russia

**Keywords:** Influenza virus, Universal vaccine, M2e ectodomain, HA2, Flagellin, Recombinant protein

## Abstract

**Background:**

Current influenza vaccines are mainly strain-specific and have limited efficacy in preventing new, potentially pandemic, influenza strains. Efficient control of influenza A infection can potentially be achieved through the development of broad-spectrum vaccines based on conserved antigens. A current trend in the design of universal flu vaccines is the construction of recombinant proteins based on combinations of various conserved epitopes of viral proteins (M1, M2, HA2, NP). In this study, we compared the immunogenicity and protective action of two recombinant proteins which feature different designs and which target different antigens.

**Results:**

Balb/c mice were immunized subcutaneously with Flg-HA2–2-4M2ehs or FlgSh-HA2–2-4M2ehs; these constructs differ in the location of hemagglutinin’s HA2–2(76–130) insertion into flagellin (FliC). The humoral and T-cell immune responses to these constructs were evaluated. The simultaneous expression of different M2e and HA2–2(76–130) in recombinant protein form induces a strong M2e-specific IgG response and CD4+/ CD8+ T-cell response. The insertion of HA2–2(76–130) into the hypervariable domain of flagellin greatly increases antigen-specific T-cell response, as evidenced by the formation of multi-cytokine-secreting CD4+, CD8+ T-cells, Tem, and Tcm. Both proteins provide full protection from lethal challenge with A/H3N2 and A/H7N9.

**Conclusion:**

Our results show that highly conserved M2e and HA2–2(76–130) can be used as important targets for the development of universal flu vaccines. The location of the HA2–2(76–130) peptide’s insertion into the hypervariable domain of flagellin had a significant effect on the T-cell response to influenza antigens, as seen by forming of multi-cytokine-secreting CD4+ and CD8+ T-cells.

**Electronic supplementary material:**

The online version of this article (10.1186/s12929-018-0433-5) contains supplementary material, which is available to authorized users.

## Background

Vaccination is the most effective way to reduce the damage caused by influenza epidemic and pandemic. Current influenza vaccines are mainly strain-specific and have limited efficacy in preventing new, potentially pandemic, influenza strains. The effective control of influenza A infection may potentially be achieved through the development of a broad-spectrum vaccine based on conserved antigens. Recent studies show that conserved influenza viral proteins (such as HA2, M2, NP and M1) can provide protective immune response against a range of influenza virus subtypes.

A number of candidate vaccines based on the ectodomain of the M2 protein (M2e) were designed, and they showed an ability to induce a robust M2e-specific humoral response and an ability to provide animals with full protection against influenza A virus challenge [1–6]. Safety and immunogenicity was shown in human as well [2, 7, 8]. Antibody-dependent-, natural killer cell- and alveolar macrophage-mediated cytotoxicities were found to be important for protection by M2e-based vaccination [9–12].

In that way, it was shown that vaccines based on conserved HA2 regions can possess prophylactic efficacy [13–15]. Currently, a search is being conducted to identify the most promising influenza A virus HA2 epitopes (amino acid 38–59, 23–185, 1–172, 76–103, 76–130, 35–107, 90–105) and recombinant proteins are being constructed based on such epitopes [14, 16–22]. Investigations in mice have shown that such recombinant proteins induce humoral and T-cell immune responses and provide protection from homologous and heterologous viruses of one phylogenetic group.

Neither immunization with traditional vaccines, nor natural influenza infection, stimulates the production of significant amounts of anti-HA2 or anti-M2e antibodies [9, 23–25]. That is due to its low immunogenicity in the presence of immunodominant receptor-binding HA1 regions. The fusion of M2e or HA2 to highly immunogenic protein carriers significantly increases their immunogenicities.

Flagellin (FliC) is a natural ligand of Toll-like receptor (TLR) 5 and represents an appropriate platform for the development of recombinant vaccines against various pathogens of viral and bacterial origin. Flagellin exhibits a strong adjuvant property when administered together with foreign antigens by parenteral, mucosal, or subcutaneous routes [26, 27]. The ability of FliC to serve both as a platform and an adjuvant for vaccine development at the same time has been demonstrated for various model infections including influenza [1, 28–33]. These studies have shown that heterologous peptides could be linked to the N- and C-terminus of flagellin, or be inserted into the hypervariable region, without disturbing the ability of FliC to bind TLR5. Previous experiments have shown that replacement of the central variable region of flagellin with M2e or HA1 did not result in the impairment of innate TLR signaling and largely improved M2e immunogenicity [30, 34, 35].

A current trend in the design of universal flu vaccines is the construction of fusion proteins based on the combination of various conserved epitopes of viral proteins. A number of studies have demonstrated the possibilities and effectiveness of this approach [19, 36, 37].

In this study, we compared two proteins based on flagellin with two targeted antigens, M2e and HA2–2(76–130), in terms of immunogenicity and protection provided. We designed a recombinant protein (Flg-HA2–2-4M2ehs) wherein a conserved fragment of HA2–2(76–130) from influenza A virus group 2 and 4 tandem copies of M2e were sequentially linked to the C-terminus of flagellin. In the second protein (FlgSh-HA2–2-4M2ehs), the HA2–2(76–130) was inserted into the hypervariable domain of flagellin and 4 tandem copies of M2e were linked to its C-terminus. The goals of this study were: to compare humoral and specific CD4+, CD8+ T-cell responses; to follow the formation of effector and central memory T-cells; and to measure the protection activity of these two proteins in a mouse model.

## Methods

### Selection of a conserved HA2(76–130) region from influenza a virus phylogenetic group 2

A search for amino acid sequences for analysis was carried out using the GenBank and GISAID databases. In order to construct consensuses, sequences were aligned using the MAFFT server using either FFT-NS-I or FFT-NS-2 algorithms (depending on the number of sequences) [38] and analyzed using Unipro UGENE (v.1.14.0) software [39]. Alignment and sequence analysis were performed using Vector NTI (v10.0) software (Invitrogen, USA). A search for experimental B-cell and CD4+ T-cell epitopes homologous to HA2 fragments was performed in the Immune Epitope Database [40]. A search for possible CD8+ T-cell epitopes was conducted using the NetCTLpan 1.1 Server [41] with default search parameters. Three-dimensional protein structures were visualized using the Chimera (1.5.3) program [42]. The Phyre2 open web resource was used for primary sequence homology simulation of protein three-dimensional structure [43].

### Construction of expression vectors

The pQE30 plasmid (Qiagen) was used to construct vectors for the expression of fusion proteins comprising: four copies of the M2e peptide (two copies M2e consensus among human influenza viruses A – M2eh and two copies of M2e from A/California/07/09 H1N1pdm09 – M2es); HA2–2(76–130) of phylogenetic group 2 influenza viruses; and flagellin from *Salmonella typhimurium*. In the first hybrid protein (Flg-HA2–2-4M2ehs), the HA2–2 fragment was linked to the C-terminus of flagellin, followed by 4 copies of M2e peptide. In the second protein (FlgSh-HA2–2-4M2ehs) the hypervariable region of flagellin was replaced by the HA2–2(76–130) fragment, and 4 copies of the M2e peptide were attached to flagellin’s C-terminus. The creation of chimeric genes was accomplished via standard genetic engineering methods. Flagellin PCR product was obtained via amplification of *Salmonella typhimurium* genomic DNA and cloned. Nucleotide sequences encoding the HA2–2(76–130) consensus sequence and tandem copies of M2e were synthesized in vitro. In this manner, two recombinant protein expression vectors (pQE30_Flg_HA2–2_4M2e and pQE30_FlgSh_HA2–2_4M2e) were created.

### Expression and purification of recombinant proteins

For recombinant proteins expression, the corresponding vectors were introduced into *E. coli* DLT1270. *E. coli* strains transformed with the pQE30_Flg_HA2–2_4M2ehs, pQE30_FlgSh_HA2–2_4M2ehs, or pQE30_FliC vectors were cultured in LB medium supplemented with ampicillin, and expression was induced by adding IPTG (1 mM final). Cells were then treated with lysozyme, and recombinant proteins were purified from the cell lysate using metal affinity chromatography; a Ni-sorbent, equilibrated with 20 mM phosphate buffer (pH 8.0) and containing 5 mM imidazole, was incubated for 60 min. Following binding of the target protein, the resin was washed with 20 mM phosphate buffer (pH 8.0) containing 20 mM imidazole. The recombinant proteins were eluted with 20 mM phosphate buffer (pH 8.0) containing 0.5 M imidazole and dialysed against 10 mM phosphate buffer (pH 7.2).

### SDS-PAGE and western blot analysis

Proteins were separated in a 12% SDS-PAGE gel (TGX Stain-Free™ Fast Cast™ Acrylamide Kit, Bio-Rad, USA) and either visualized by staining with Coomassie G-250 or electro-transfered to a nitrocellulose membrane (Bio-Rad, USA). We used molecular weight markers from 15 to 250 kDa (Precision Plus Protein™ Dual Xtra Standarts, Bio-Rad, USA). Membranes were blocked with 3% BSA overnight at room temperature and protein bands were detected by membrane staining with rabbit polyclonal antibodies specific to bacterial flagellin (Abcam, UK) or mouse anti-M2e monoclonal antibody 14C2 (Abcam, UK); incubations with antibody were performed for 1 h in PBS containing 0.1% Tween-20 and 3% BSA and then washed. Bands were visualized by staining the membrane for 1 h at room temperature with peroxidase labeled secondary antibodies, namely goat anti-rabbit IgG (Invitrogen, USA) or goat anti-mouse IgG (Abcam, UK). Finally, blots were incubated in TMB Immnublot solution (Invitrogen, USA) for 5 min.

### Flagellin bioactivity assay

Flagellin bioactivity was analyzed using HEK-Blue™hTLR5 cells (InvivoGen, USA) according to the manufacturer’s instruction (InvivoGen, USA). A suspension of fresh HEK-Blue™-hTLR5 or HEK-Blue Null 1 cells were seeded at a density of 450,000 cells/ml onto 96-well plates (Nunc, Thermo Scientific, Denmark). The cells were immediately stimulated with one of the following recombinant proteins: Flg-HA2–2-4M2ehs; FlgSh-HA2–2-4M2ehs; FliC; FLA-ST (positive control, InvivoGen USA); or ODN2006 (negative control, InvivoGen, USA). After 24 h, NF-κB-induced SEAP activity was assessed using QUANTI-Blue™ and an optical density (OD) reading at 655 nm using an i-Mark microplate reader (Bio-Rad). The mean OD of stimulated cells was subtracted from the mean OD of unstimulated cells.

### Mouse immunization

Female BALB/c mice (16-18 g) were purchased from the Stolbovaya mouse farm at the State Scientific Center of Biomedical Technologies, Russian Academy of Medical Sciences. Animals were kept at the vivarium of the Research Institute of Influenza in accordance with working regulations. Mice were immunized subcutaneously (s.c.) on days 0 (primary vaccination), 14 (first boost), and 28 (second boost) with 10 μg/0.1 ml of either Flg-HA2–2-4M2ehs or FlgSh-HA2–2-4M2ehs. Control mice were injected (s.c.) with 0.1 ml of PBS or 10 μg/0.1 ml of recombinant FliC.

### Collection of mouse sera and splenocytes

Blood samples were obtained 2 weeks after the second boost, following euthanasia in a CO_2_-chamber (Vet Tech Solutions, UK). In order to obtain serum, blood samples were incubated at 37 °C for 30 min. After blood clot formation, samples were cooled on ice for 1 h, followed by centrifugation at 400 g for 15 min. Serum aliquots (30 μL) from 6 mice of each group were frozen at − 20 °C.

Mouse splenocytes were prepared according to the BD Pharmingen™ protocol. Mice from experimental and control groups (5 mice from each group) were euthanized (CO_2_-chamber) on the 14th day after second boost. Mouse spleens were removed aseptically, homogenized using a Medimachine (BD Biosciences, USA), and purified from cell debris by filtration through a syringe filter with a 70 μm pore size (Syringe Filcons, BD Biosciences, USA). Erythrocytes were lysed with ACK lysing buffer (0.15 M NH_4_Cl, 1.0 M KHCO_3_, 0.1 mM Na_2_EDTA, pH 7.2–7.4). Splenocytes were washed with RPMI-1640 complete medium containing 10% FBS, 2 mM L-glutamine, 100 U/mL penicillin, and 100 μg/ml streptomycin. The cell concentrations were adjusted to 5 × 10^6^ cells/ml.

### Synthetic peptides

The following peptides were experimentally tested (synthesized by Verta, Russia):

- M2es SLLTEVETP**T**R**S**EW**E**CRC**S**DSSD (M2e of A/California/07/09 H1N1pdm09).
- M2eh SLLTEVETP**I**R**N**EW**G**CRC**N**DSSD (consensus M2e in human influenza A viruses).

Residues that differ between the sequences are displayed in bold font and underlined.

### Antibody detection in the sera

Antigen-specific IgG levels were determined by ELISA in 96-well microtiter plates (Greiner, Germany) coated overnight at 4 °C with the M2e peptides (5 μg/ml) in PBS or purified viruses (2 μg/ml) (A/Aichi/2/68(H3N2), A/Shanghai/2/2013-PR8-IDCDC(H7N9)) in PBS pH 7.2 as previously described [5]*.* Polyclonal HRPO-labeled goat anti-mouse IgG, IgG1, IgG2a, IgG2b, and IgG3 antibodies (Abcam, UK) were used. TMB (BD Bioscience, USA) was used as a substrate; the incubation time was 15 min. The OD was measured using an i-Mark microplate reader (Bio-Rad) at a wavelength of 450 nm. The maximal serum dilution that had an optical density at least 2 times higher than twice mean value of the blank was taken as the titer.

### Intracellular cytokine staining (ICS) assay

Multiparameter flow cytometry was performed in accordance to BD Pharmingen™ Protocol. In brief, splenocytes were harvested at day 14 post second boost; 5 × 10^6^ cells were stimulated in a Nunc™ 96-well conical-bottom plate (Thermo Scientific™, USA) for 6 h at 37 °C with 10 μg of M2eh peptide and 1 μg of influenza virus A/Aichi/2/68 (H3N2) in the presence of 1 μg/ml of Brefeldin A (BD Bioscience, USA) and purified hamster anti-mouse CD28. The cells were then washed, and Fc receptors were blocked using CD16/CD32 antibodies (Mouse BD Fc Block, BD Pharmingen, USA) for 30 min. Next, cells were incubated with Zombie Aqua (Zombie Aqua™ Fixable Viability Kit, Biolegend, USA) to enable gating of live cells during analysis and subsequently stained with antibodies (CD3e-FITC, CD8a-APC-Cy7, CD4-PerCP, CD62L-PE-Cy7, CD44-APC) at 4 °C for 30 min (antibodies from BD Pharmingen, USA). Cells were permeabilized using the BD Cytofix/Cytoperm Plus (BD Bioscience, USA) protocol and stained with anti-TNF-α-BV421 or anti-IFN-γ-PE (BD Pharmingen, USA). Sample acquisition (50,000 live CD3+ were collected) was performed with a BD FACS Canto II flow cytometer (Becton Dickinson, USA) and analyzed using Kaluza, version 1.5 (Beckman Coulter, USA).

### Viruses

In this study, we used influenza strains A/Aichi/2/68 (H3N2), A/Shanghai/2/2013-PR8-IDCDC (H7N9), A/Chicken/Kurgan/05/05 RG (H5N1), and A/California/07/09 (H1N1pdm09) received from the Collection of Influenza and Acute Respiratory Viruses at the Research Institute of Influenza. Influenza strains A/California/07/09(H1N1pdm09) and A/Shanghai/2/2013-PR8-IDCDC(H7N9) were received from C.D.C. (Atlanta, USA). Influenza virus A/Kurgan/05/05 RG(H5N1) is a non-virulent strain obtained by the Research Institute of influenza using methods of reverse genetics [5]. Influenza viruses A/Aichi/2/68 (H3N2) and A/Shanghai/2/2013-PR8-IDCDC (H7N9) are mouse-adapted viruses obtained by the Research Institute of influenza by serial mouse/egg passages; its M2, NA, and HA amino acid sequences have remained identical to the original virus, but it has acquired the ability to lethally infect mice ([5], Additional file 1). Experimental work with influenza strains was carried in a BSL2 facility.

### Influenza virus challenge in mice

In lethal challenge experiments, we used the A/Aichi/2/68 (H3N2) and A/Shanghai/2/2013-PR8-IDCDC (H7N9) mouse-adapted influenza strains. Two weeks after the final immunization, mice (*n* = 10/group) were challenged intranasally (i.n.) with 10 times the LD_50_ doses (10LD_50_) of A/Aichi/2/68 (H3N2), or A/Shanghai/2/2013(H7N9)-PR8-IDCDC. Mice were anesthetized under inhalation anesthesia (2–3% isoflurane mixed with 30% oxygen (O_2_) and 70% nitrous oxide (N_2_O)). Mice previously administered PBS or recombinant FliC were challenged as negative controls. The animals were monitored for survival and weight loss daily for 2 weeks.

### Lung virus titers

Two weeks after the final immunization, mice were challenged (i.n.), as follows: A/Aichi/2/68 (H3N2) at 10LD_50_; A/Shanghai/2/2013 (H7N9)-PR8-IDCDC at 10LD_50_; A/Chicken/Kurgan/05/05 RG (H5N1) at 100MID; or A/California/07/09 H1N1pdm09 at 100MID. Five mice from each group were sacrificed (CO_2_-chamber, as described above) on day 6 post-infection, and the lungs were removed as previously described [5]. In 96-well cell-culture plates, ten-fold serial dilutions of samples, in quadruplicate, were added to monolayers of MDCK cells (in serum-free medium containing 2 μg/ml of TPCK-trypsin (Sigma)) and incubated for 72 h. Viral cytopathic effects were monitored daily, and the viral titers were determined by HA test with 0.5% chicken erythrocytes. The viral titer was calculated by the Reed and Mench method and expressed as log 50% tissue culture infectious dose (TCID_50_).

### Statistical analysis

Differences in antibody levels, antigen-specific cytokine producing T-cells, and viral titers in lung suspensions were evaluated by the Mann-Whitney U-test. Significant differences in survival among mouse groups were analyzed by the Montel-Cox test. If a *P* value was less than 0.05, the difference was considered to be significant. The analysis was done by using GraphPad Prism, version 6.0.

## Results

### Conserved sequences of М2 and НА2

**Fig. 1:**
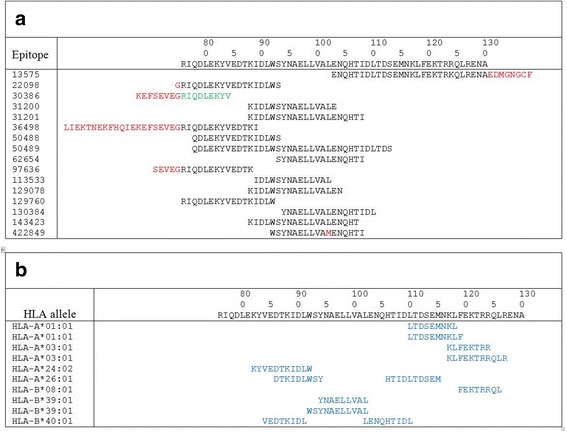
**a** Experimental B-cell and CD4+ Т-cell epitopes, homologous to the consensus НА2 (76–130) fragment by more than 90%. IEDB database search result is presented; non homologous amino acids are marked with red. Green font identifies the single B-cell epitope. Black font identifies the CD4+ T-cells epitopes **b** Potential CD8+ Т-cell epitopes inside HA2(76–130) fragment for a representative set of alleles; results of analysis using NetCTLpan1.1 Server is shown. Blue font identifies the CD8+ T-cells epitopes

Conserved fragments of M2 protein and the second subunit of hemagglutinin (HA2) from influenza A viruses were selected as a target antigens: consensus M2e of human influenza A viruses (M2eh); M2e of A/California/07/09 H1N1pdm09 (M2es); and HA2(76–130) consensus for influenza viruses of 2 phylogenetic group A/H3N2 and A/H7N9 (HA2–2) (Table 1). Fragment НА2–2(76–130) represents the main (long) α-helix of hemagglutinin’s second subunit; the fragment is partially accessible on the molecule’s surface. The identity of consensus HA2 sequences in influenza phylogenetic group 2 (subtypes Н3 and Н7) in the frame (76–130) is 63.6%. In analysis which considers amino acids with similar physicochemical characteristics to be a match, the homology of the HA2(76–130) fragment reaches up to 80% (Additional file 2). The single B-cell epitope and most experimental СD4+ T-cell epitopes are located in the first half of the fragment (Fig. 1а, Additional file 3). Theoretical CD8+ Т-cell epitopes of various HLA-alleles are also present in the described fragment (Fig. 1b).

**Table 1:** Amino acid sequences of conserved fragments of M2 and HA2–2 fragments from human and avian influenza A viruses

Epitopes	Amino acid sequence
consensus M2e of human influenza A viruses	SLLTEVETPIRNEWGCRCNDSSD
M2e of A/California/07/09 (H1N1pdm09)	SLLTEVETP**T**R**S**EW**E**CRC**S**DSSD
M2e of A/Aichi/2/68 (H3N2)	SLLTEVETPIRNEWGCRCNDSSD
M2e of A/Shanghai/2/2013 -PR8-IDCDC(H7N9)	SLLTEVETP**T**R**TG**W**E**C**N**C**SG**SS**E**
HA2–2(76–130) consensus for influenza viruses A/H3N2	**RIQDLEKYVEDTKIDLWSYNAELLVALENQHTIDLTDSEMNKLFE** ***R*** **TR** ***K*** **QLRENA**
HA2–2(76–130) consensus for influenza viruses A/H7N9 from birds and humans	***Q*** **I** ***GNVINWTR*** **D** ***SMTEV*** **WSYNAELLVA** ***M*** **ENQHTIDL** ***A*** **DSEMNKL** ***Y*** **ER** ***VKR*** **QLRENA**
HA2–2-(76–130) consensus for influenza viruses A/H3N2 and A/H7N9	**RIQDLEKYVEDTKIDLWSYNAELLVALENQHTIDLTDSEMNKLFEKTRRQLRENA**

Residues that differ between the sequences are displayed in bold font and underlined

### Design and characterization of fusion proteins

**Fig. 2:**
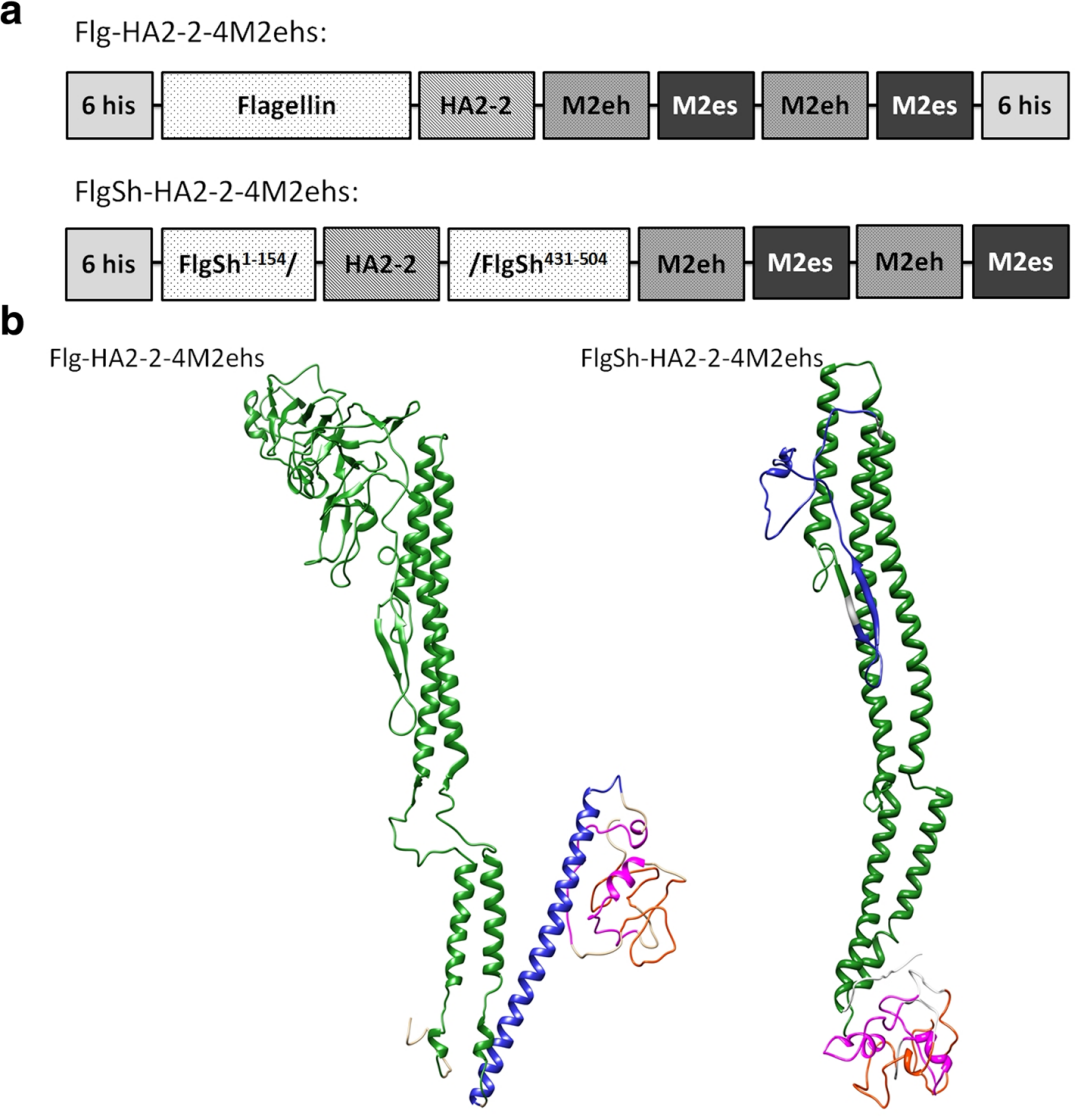
Structure (**a**) and theoretical modelling of 3D-structures (**b**) of monomeric recombinant fusion proteins Flg-HA2–2-4M2ehs and FlgSh-HA2–2-4M2ehs. Sizes of boxes are not drawn to scale. Yellow marks M2es; pink marks M2eh; blue marks HA2 fragment; and green is flagellin. Modelling performed with Phyre2 server, visualization made with USCF Chimera

Fusion protein Flg-HА2–2-4M2ehs was designed containing full-length flagellin from *S. typhimurium* with the histidine tag at the N-terminus, the HA2–2 consensus sequence (76–130) of influenza A virus phylogenetic group 2 (A/H3N2, A/H7N9), and 4 tandem copies of M2e (М2h-М2s-М2h-М2s) at the C-terminus (Fig. 2a). In fusion protein FlgSh-HА2–2-4M2ehs, the central variable domain of flagellin was replaced with consensus HA2–2 fragment (76–130), and 4 tandem copies of M2e (М2h-М2s-М2h-М2s) were linked to the C-terminus (Fig. 2a). Flexible, glycine-rich (GGGSG) linkers were inserted between M2e peptide sequences. Three-dimensional structure of Flg-HА2–2-4M2ehs demonstrated the preservation of the α-helix structure in HA2–2(76–130) (Fig. 2b), suggesting that most of the native structure was preserved. The insertion of HA2–2 into hypervariable domain of flagellin showed only partial retention of α-helix structure in HA2–2(76–130). Both recombinant proteins conserved the native flagellin structure.

**Fig. 3:**
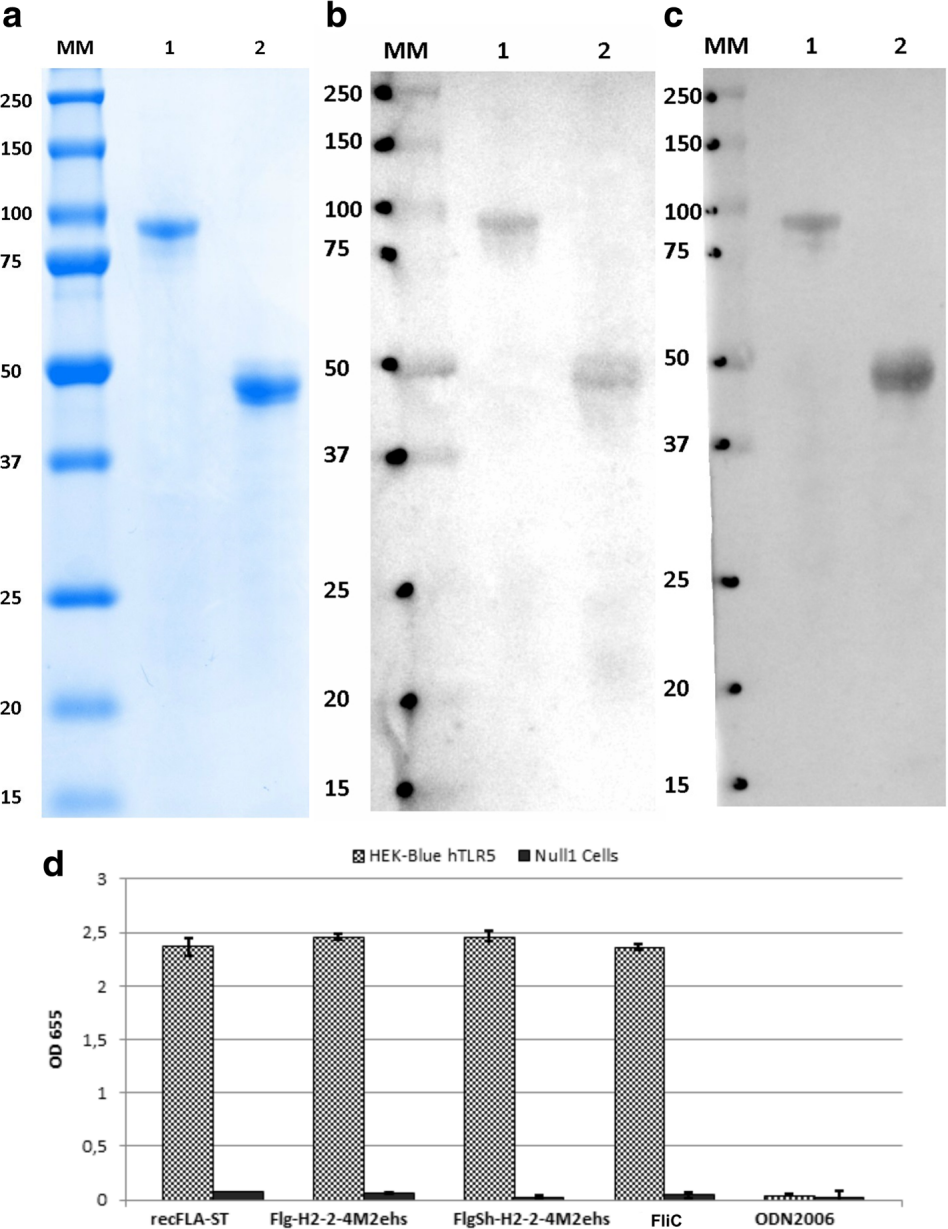
SDS-PAGE (**a**) Coomassie brilliant blue staining and Western blotting analysis of recombinant fusion proteins Flg-HA2–2-4M2ehs, FlgSh-HA2–2-4M2ehs by anti-M2e mAb 14C2 **(b**) and anti-Flg mAb 93,713 (**c**); immunostaining with HRP-conjugated second antibodies and TMB substrate. Positions of molecular weight markers (MM) are indicated. 1 - Flg-H2–2-4M2ehs; 2 – FlgSh-H2–2-4M2ehs. **d** Response of HEK-Blue™ hTLR5 cells to recombinant proteins. HEK-Blue™ hTLR5 and HEK-Blue™ Null1 (control) cells were incubated in HEK-Blue™ Detection medium and stimulated with 100 ng/ml recFLA-ST (positive control), 100 μg/ml ODN2006 (negative control) and 100 ng/ml of FliC, Flg-HA2–2-4M2ehs, or FlgSh-HA2–2-4M2ehs. After 24 h incubation, the levels of NF-kB-induced SEAP were determined by reading the OD at 655 nm. Error bars represent the s.d. obtained from three biological replicates

The purity of the Flg-HA2–2-4M2ehs and FlgSh-HА2–2-4М2еhs recombinant proteins was evaluated by SDS-PAGE which indicated single bands with molecular weights of approximately 74 kDa and 45 kDa, respectively, the gel indicated approximately 95% purity (Fig. 3a). The identity and integrity of the recombinant proteins were estimated by Western blot analysis with antibodies specific to flagellin (Fig. 3b) and 14C2 mAb to M2e (Fig. 3c). The results confirmed the presence of flagellin and M2e in both of the purified proteins.

In order to demonstrate that recombinant proteins based on full-length and truncated flagellin (Flg-HA2–2-4M2ehs, FlgSh-HA2–2-4M2ehs) can induce immune response via TLR5, we used the HEK-Blue™ hTLR5 cell line which expresses human TLR5 (Fig. 3d) and estimated the levels of NF-kB-induced SEAP activity. Both fusion proteins induced strong TLR5-mediated signaling in this cell line. The magnitude of the response was comparable to that of the established TLR5-specific ligand FLA-ST (flagellin from *S. typhimurium)*.

### HA2-M2e fusion proteins induced a strong IgG response

**Fig. 4:**
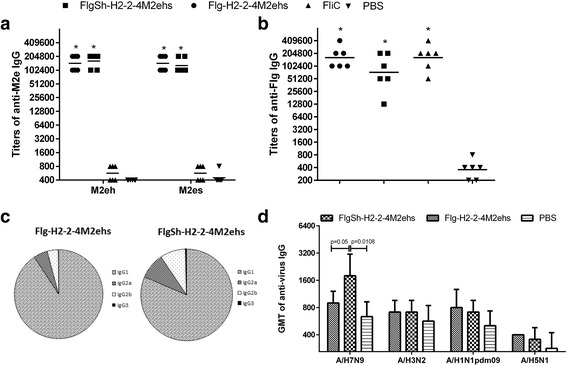
Antibody response in serum. BALB/c mice (*n* = 6/group) were immunized s.c. with 10 μg of Flg-HA2–2-4M2ehs, or FlgSh-HA2–2-4M2ehs on days 0, 14, 28. Control groups mice were administered with FliC (10 μg) and PBS. Two weeks post second boost, M2e-specific IgG responses (**a**) and FliC-specific IgG responses (**b**) were evaluated by ELISA. **c** Anti-M2e IgG subclasses tested against M2e peptide in serum, 2 weeks post second boost, were determined by ELISA. **d** Titers of serum IgG to influenza viruses A/H7N9, A/H3N2, A/H1N1pdm09, A/H5N1. Data are presented as the geometric mean titers (GMT) with 95% CI (confidence interval). Statistical significance was determined using Mann-Whitney U-test. The *P* values between immunized and control group are indicated. *significant difference from control groups, *p* < 0.01

The immunogenicity of the Flg-HA2–2-4M2ehs and FlgSh-HA2–2-4M2ehs fusion proteins was examined in Balb/c mice immunized (s.c.) three times. Two week post second boost, mice were bled, and sera from 6 mice were tested individually for anti-M2e IgG, IgG1, IgG2a, IgG2b, and IgG3 by ELISA. A strong, specific immune response was induced post-second boost (Fig. 4a). Induced antibodies efficiently bound to synthetic M2eh and M2es peptides, corresponding to M2e sequences in the recombinant proteins. No significant differences were found in the levels of anti-M2eh and anti-M2es IgG between recombinant proteins. It was shown also (Fig. 4b) that the level of IgG to FliC was 2-fold lower in the FlgSh-HA2–2-4M2ehs group than in mice immunized with full-length proteins (Flg-HA2–2-4M2ehs and FliC).

We also examined the profile of M2e-specific IgG subclasses after the second boost (in mice) with Flg-HA2–2-4M2ehs, or FlgSh-HA2–2-4M2ehs (Fig. 4c). Both proteins stimulated predominantly anti-M2e IgG1 production, but the levels of IgG2a and IgG2b were 2-fold higher after immunization with FlgSh-HA2–2-4M2ehs than with of Flg-HA2–2-4M2ehs.

The selected HA2–2 consensus sequence (76–130) is highly conserved in phylogenetic group 2 influenza viruses (80% homology). Therefore, it was important to evaluate the formation of virus-specific antibodies after immunization of mice with the two recombinant proteins. According to ELISA, induced HA2–2-specific IgG bound to influenza virus from phylogenetic group 2 (A/H7N9) (Fig. 4d), and the titers of virus-specific IgG were significantly higher in mice immunized with FlgSh-HA2–2-4M2ehs than with Flg-HA2–2-4M2ehs. In addition, induced antibodies bound to hemagglutinin under native conformation (pH 7.2), which indicates accessibility of the HA2–2 target sequence on the virion’s surface. However, the ability of antibodies to bind with other influenza subtypes (A/H3N2, A/H1N1pdm09, A/H5N1) in mice immunized with the Flg-HA2–2-4M2ehs or FlgSh-HA2–2-4M2ehs fusion proteins was low.

### HA2-M2e fusion proteins activated antigen-specific T-cell response in spleen

In order to test antigen-specific T-cell responses, splenocytes from s.c immunized (Flg-HA2–2-4M2ehs, or FlgSh-HA2–2-4M2ehs) and from control (PBS) mice (5 mice in each group), two week post second boost, were assayed against M2e peptide and influenza virus A/Aichi/2/68 (H3N2) using ICS. Additional file 4: (Figure A) shows the gating strategy for single or double cytokine-secreting antigen-specific CD4+, CD8+ T-cells, effector memory T-cells (Tem, CD44+/CD62L^−^), and central memory T-cells (Tcm, CD44 + CD62L+).

#### M2e-specific T-cell response

**Fig. 5:**
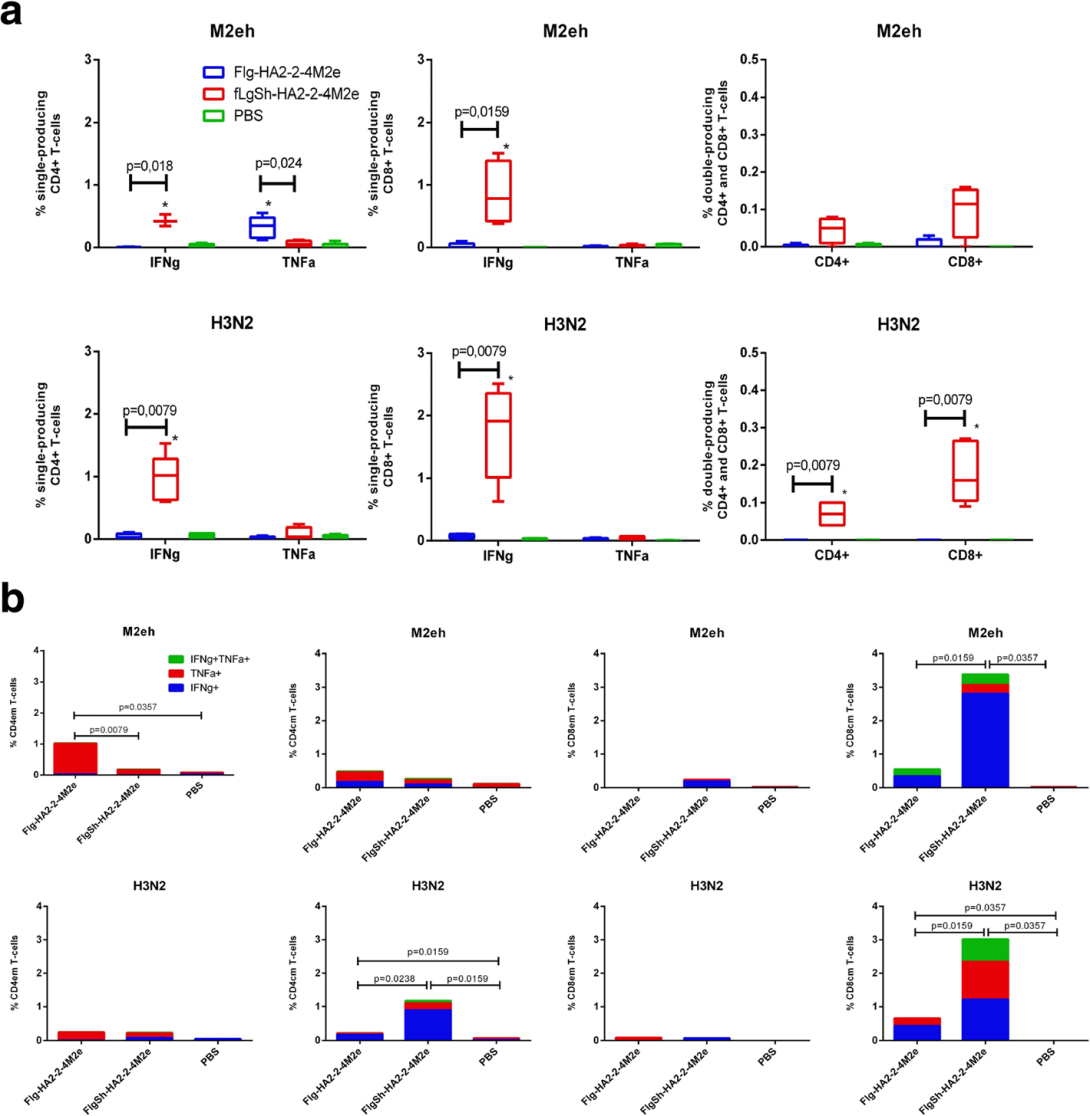
Specific T-cell response in spleen. BALB/c mice (*n* = 5/group) were immunized s.c. with 10 μg of Flg-HA2–2-4M2ehs or FlgSh-HA2–2-4M2ehs on days 0, 14, 28. Splenocytes were isolated from 5 mice of each group at day 14 post-second boost and assayed for M2e-specific and A/H3N2-specific CD4+ and CD8+ T-cell responses. **a** Antigen-specific CD4+ and CD8+ T-cells. **b** Antigen-specific Tem and Tcm cells. Data are presented as the mean ± SEM. Statistical significance was determined using the Mann-Whitney U-test. The *P* values between groups are indicated. *significant difference from control group, *p* < 0.02

On average, 0.32% CD4+ T-cells from Flg-HA2–2-4M2ehs mice, versus 0.03% CD4+ T-cells from control mice and 0.046% in mice treated with FlgSh-HA2–2-4M2ehs, secreted TNF-α following splenocyte stimulation with M2e peptide (Fig. 5a). In mice immunized with FlgSh-HA2–2-4M2ehs, we observed M2e-specific CD4+ and CD8+ T-cells secreting IFN-γ (on average 0.43 and 0.87%, respectively); both the control group (0.03 and 0.0%) and the Flg-HA2–2-4M2ehs group (0.02 and 0.08%) showed lower levels. These results suggest that of immunization of mice with both recombinant proteins induces a strong activation of single cytokine producing (TNF-α + or IFN-γ+) M2e-specific CD4+ and/or CD8+ T-cells.

#### H3N2-specific T-cell response

The effects of splenocyte stimulation with the A/Aichi/2/68 (H3N2) influenza virus were as follows (Fig. 5a). After immunization with Flg-HA2–2-4M2ehs, a significant amount of virus- specific, cytokine producing CD4+ and CD8+ T-cells were not formed. Immunization with FlgSh-HA2–2-4M2ehs, in mice, lead to the generation of virus-specific CD4+ and CD8+ T-cells producing IFN-γ (0.97 and 1.73% respectively). This contrasts with lower numbers in the control group (0.06 and 0.07%) and lower numbers in the Flg-HA2–2-4M2ehs group (0.04 and 0.07%). In addition, FlgSh-HA2–2-4M2ehs immunization induced double cytokine producing (TNF-α + IFN-γ+) A/H3N2-specific CD4+ and CD8+ T-cells (0.07 and 0.18%) versus control (0%) and the Flg-HA2–2-4M2ehs group (0%). The recombinant protein featuring a partially replaced flagellin (FlgSh-HA2–2-4M2ehs) induces virus-specific CD4+ and CD8+ T-cell responses in the form of multiple cytokine producers (TNF-α + IFN-γ+) and also single cytokine producers (IFN-γ+).

#### M2e-specific CD4 + and CD8+ memory T-cells

At two weeks after s.c vaccination, effector memory (Tem, CD44 + CD62L^−^) and central memory (Tcm, CD44 + CD62L+) CD4+ and CD8+ T-cells were determined in mouse spleen (Fig. 5b, Additional file 4: Figure B, C, D). Low frequencies of M2e-specific CD4+ Tem and Tcm (0.17 and 0.22%, respectively) were measured in the FlgSh-HA2–2-4M2ehs group compared with PBS (0.085 and 0.1%). After immunization with Flg-HA2–2-4M2ehs, 1.02% of M2e-specific Tem and 0.46% Tcm was detected (Fig. 5b). M2e-specific CD4+ Tem and Tcm cells in the Flg-HA2–2-4M2ehs group were predominantly single-producers of either TNF-α or IFN-γ. M2e-specific CD8+ Tem cells were detected only in the FlgSh-HA2–2-4M2ehs group (0.19%) and were predominantly single-producers of IFN-γ. The number of M2e-spesific CD8+ Tcm cells significantly increased in the FlgSh-HA2–2-4M2ehs group (on average 3.38%) in comparison to the Flg-HA2–2-4M2ehs (0.34%) and PBS group (0.02%) groups. M2e-specific CD8+ cells in the FlgSh-HA2–2-4M2ehs and Flg-HA2–2-4M2ehs groups were predominantly single-producers of IFN-γ + (2.81 and 0.28%, respectively).

#### H3N2-specific CD4 + and CD8+ memory T-cells

The measured amounts of virus-specific CD4+ Tem cells (Fig. 5b, Additional file 4: Figure. B, C, D) were virtually identical between the Flg-HA2–2-4M2ehs and FlgSh-HA2–2-4M2ehs groups (0.25 and 0.22%, respectively), in contrast to PBS (0.05%). The population was predominantly single-producing TNF-α + CD4+ cells. The amount of virus-specific CD4+ Tcm cells in the FlgSh-HA2–2-4M2ehs group was significantly higher (1.17%) than in the Flg-HA2–2-4M2ehs (0.26%) and PBS (0.048%) groups. In the FlgSh-HA2–2-4M2ehs group, the dominant cell type was single-producing IFN-γ + cells (0.89%), yet CD4+ Tcm single-producing TNF-α + (0.21%) and double-producing TNF-α + IFN-γ + cells (0.074%) were also identified. In the Flg-HA2–2-4M2ehs group, the number of virus-specific CD4+ Tcm was significantly higher than in the control group, and single-producing IFN-γ + types were dominant (0.17%).

Virus-specific CD8+ Tem cells were measured in low frequencies in the Flg-HA2–2-4M2ehs and FlgSh-HA2–2-4M2ehs groups. Virus-specific CD8+ Tcm cells, however, were measured in high frequencies in both groups compared with PBS. Virus-specific CD8+ Tcm cells showed the following pattern, on average: 3.02% in the FlgSh-HA2–2-4M2ehs group; 0.43% in the Flg-HA2–2-4M2ehs group; and 0.025% in the control mouse group. Immunization with FlgSh-HA2–2-4M2ehs led to the formation of single-producing TNF-α + (1.13%), IFN-γ + (1.21%), and double-producing TNF-α + IFN-γ + (0.68%) virus-specific CD8+ Tcm cells (Fig. 5b, Additional file 4: Figure. B, C, D). On the other hand, only single-producing TNF-α + (0.22%), IFN-γ + (0.43%) and a lack of double-producing cytokine A/H3N2-specific CD8+ Tcm were detected in the Flg-HA2–2-4M2ehs group.

Altogether, these results suggest that FlgSh-HA2–2-4M2ehs induces the strong formation of M2e-specific single-(IFN-γ+) and double-producing (IFN-γ + TNF-α+) CD4+ and CD8+ cells. Flg-HA2–2-4M2ehs induces the formation of only M2e-specific single-producing (TNF-α+) CD4+ types. In addition, s.c immunization with Flg-HA2–2-4M2ehs dominantly induced M2e-specific single-producing TNF-α + CD4+ Tem cells in mouse spleen; this is unlike the FlgSh-HA2–2-4M2ehs group in which we detected a high number of single-producing IFN-γ + CD8+ Tcm cells.

A virus-specific T-cell response was characterized by high frequencies of A/H3N2-specific single-(IFN-γ+) and double-producing (IFN-γ + TNF-α+) CD4+ and CD8+ types only in FlgSh-HA2–2-4M2ehs. In this group, we also documented the formation of A/H3N2-specific CD4+ and CD8+ T-cells with central memory cells markers (CD44 + CD62L+); these types were seen as single-producers (IFN-γ+, or TNF-α) and as double-producers (IFN-γ + TNF-α+).

### Immunization with HA2-M2e fusion proteins protected mice from lethal H3N2 and H7N9 viral challenge

**Fig. 6:**
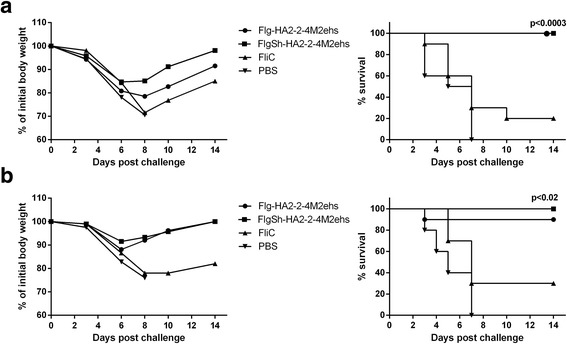
Efficacy of immunization. Groups of 10 Balb/c mice were immunized with fusion proteins Flg-HA2–2-4M2ehs and FlgSh-HA2–2-4M2ehs. Two weeks post-second boost, mice were challenged with (**a**) 10LD_50_ A/Aichi/2/68 (H3N2) or (**b**) 10LD_50_ A/Shanghai/2/2013(H7N9)-PR8-IDCDC. Body weight (left) and survival rate (right) were monitored daily during 14 days. The *P* values (Montel-Cox test) between immunized and control groups are indicated

In order to evaluate the protective efficacy of the two fusion proteins, mice were immunized (s.c.) three times, at 2-week intervals. At two week post second boost mice, mice challenged intranasally with 10LD_50_ dose of mouse-adapted viruses from phylogenetic group 2 (A/Shanghai/2/2013-PR8-IDCDC H7N9 or A/Aichi/2/68 H3N2). The morbidity and mortality of infected mice were monitored for 14 days. As shown in Fig. 6, all mice that received FlgSh-HA2–2-4M2ehs were fully protected (100% survival) from lethal A/Aichi/2/68 (H3N2) (*p* = 0.0003 from FliC group and *p* < 0.0001 from PBS group) and A/Shanghai/2/2013-PR8-IDCDC (H3N2) (*p* = 0.0014 from FliC group and *p* < 0.0001 from PBS group) virus infections; they experienced slight body weight loss (8.5–15%) and started to recover at day 6 post challenge. Mice immunized with Flg-HA2–2-4M2ehs showed 90% survival from A/Shanghai/2/2013-PR8-IDCDC (H3N2) (*p* = 0.0151 from FliC group and *p* = 0.0002 from PBS group) and full protection from A/Aichi/2/68 (H3N2) (*p* = 0.0003 from FliC group and *p* < 0.0001 from PBS group). In this group, body weight loss was 12–19%, and animals started to recover at day 6–8 post challenge. In comparison, mice mock-immunized with PBS were not protected from lethal challenge, and all died at or before day 8 post infection (Fig. 6). In mice immunized with carrier protein (FliC), the survival after lethal challenge was 20–30%. Both control groups experienced significant body weight loss (more than 20%) in the 8 day period post challenge.

### HA2-M2e fusion proteins immunization significantly reduced influenza viral load in lungs

**Fig. 7:**
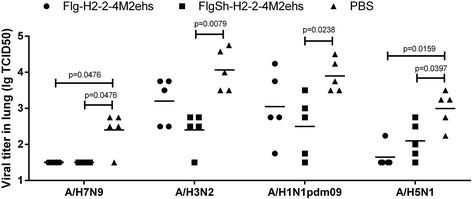
Detection of viral titers in mouse lung. Mice (*n* = 5/group) immunized with fusion proteins Flg-HA2–2-4M2ehs and FlgSh-HA2–2-4M2ehs were i.n. challenged with: A/Shanghai/2/2013(H7N9)-PR8-IDCDC (10LD_50_); A/Aichi/2/68(H3N2) (10LD_50_); A/California/07/09H1N1pdm09 (100MID); or А/Chicken/Kurgan/05/05RG(H5N1) (100MID). Viral titers were detected 6 days post challenge. The data are expressed as log TCID50. Horizontal bars indicate mean among 5 mice per group. The lower limit of detection is 0.5log TCID_50_. Statistical significance was determined using the Mann-Whitney U-test. The *P* values between immunized and control group are indicated

Two weeks after completion of the vaccination protocol, mice from test and control groups were infected (i.n.) with influenza viruses: A/Aichi/2/68 (H3N2, group 2); A/Shanghai/2/2013-PR8-IDCDC (H7N9, group 2); A/Chicken/Kurgan/05/05 RG (H5N1, group 1); or A/California/07/09 (H1N1pdm09, group 1). Six days post-challenge, five mice from each group were sacrificed for titration of residual lung virus. We observed a significant decrease in lung viral titres (Fig. 7) in mice treated with FlgSh-HA2–2-4M2ehs, compared to naïve mice, following challenge with A/Shanghai/2/2013-PR8-IDCDC(H7N9) (*p* = 0.0476), A/Aichi/2/68 (H3N2) (*p* = 0.0079), A/California/07/09 H1N1pdm09 (*p* = 0.0238), or A/Chicken/Kurgan/05/05 RG (H5N1) (*p* = 0.0397). A substantial reduction in lung viral titres in mice treated with Flg-HA2–2-4M2ehs was only observed after challenge with influenza strains A/Shanghai/2/2013-PR8-IDCDC (H7N9) (*p* = 0.0476) and А/Chicken/Kurgan/05/05 RG (H5N1) (*p* = 0.0159).

## Discussion

The cross-protective properties of M2e-based vaccines have been shown by a number of studies and are mediated mainly through humoral, rather than T-cellular, immune responses [9–11]. However, the formation of M2e-specific CD4+ T-cell response can make a significant contribution to the protection from influenza due to the fact that memory CD4+ T-cells can regulate the responses of innate immune cells, antibody-producing B-cells, and cytotoxic CD8+ T-cells, in addition to directly combating pathogens [44]. Strong virus-specific CD4+ T-cell response have been correlated with protection in humans [45]. Experimental studies of T-cell-mediated response in mice have demonstrated that influenza-specific memory CD8+ and CD4+ cells are sufficient to protect against heterosubtypic influenza challenge [46, 47]. The importance of CD8+ T-cells in the control of human influenza has been shown in various animal models [48, 49]. Recently, it was demonstrated that pre-existing virus-specific CD8+ T-cells in humans correlated with protection against A/H1N1pdm09 [50]. It was concluded that the combination of non-neutralizing antibodies with CD8 + T cells can provide complete protection against a lethal influenza infection. Thus, novel cross-protective influenza vaccines should be capable of efficiently inducing not only antibodies to conserved influenza antigens, but also cross-protective T-cell responses (CD4+ and CD8+).

We have previously indicated [5] that M2e from human and avian influenza A viruses, when linked to the highly immunogenic carrier flagellin, induced a robust anti-M2e response and provided protection against various influenza A subtypes (A/H1N1, A/H3N2, A/H2N2, A/H1N1pdm09, and H5N1). In addition, such candidate vaccine can stimulate the formation of M2e-specific Th2 IL-4-producing cells; a strong M2e specific Th1 and CD8+ response is not formed. On the other hand, insertion of M2e into the major immunodominant loop of the hepatitis B core antigen (in the presence of CpG) induced the formation of M2e-specific CD4+ and CD8+ cells producing IFN-γ [6].

Several studies have shown that immunization of mice with fusion proteins based on flagellin (flagellin-GFP, flagellin-OVA) stimulated a CD8+ response to the target antigen [51–53]. It was shown that antigen, but not the TLR5 signal, is a limiting factor in the formation of the CD8+ T-cell response [53]. Thus, flagellin may serve as a platform for vaccines containing poorly processed antigens bearing CD8+ epitopes. Recently, we reported the design of a recombinant fusion protein (FlgMH) based on flagellin and a highly conserved HA2 fragment (35–107) representing a consensus among A/H2N2 strains [20]. We also showed that the fusion of HA2(35–107) to flagellin stimulated not only a mixed Th1/Th2 response, including the formation of cross-reactive antibodies which bind to phylogenetic group 1 influenza viruses (H1, H2, H5), but also induced specific CD8+ T-cells, producing IFNγ.

Therefore, we developed a candidate vaccine design based on two conserved antigens (M2e from human influenza A viruses and HA2–2(76–130) from phylogenetic group 2 influenza viruses) linked to flagellin. In this study we compared two recombinant fusion protein variants featuring differing insertion points of HA2–2(76–130) into flagellin. The identity and integrity of both fusion constructs were validated. In addition, it was showed that both fusion proteins can induce immune responses via TLR5.

The cross-protective properties of M2e-based vaccines have been shown by a number of studies [1–8]. They have shown the important role of anti-M2e antibodies in cross-protection against influenza A viruses. Our results demonstrate the ability of both fusion proteins with two target antigens (M2e and HA2–2) to induce high titers of serum anti-M2e IgG capable of binding to synthetic M2eh and M2es peptides at a similar level. The profile of M2e-specific IgG subclasses, seen after mouse immunization, showed that both proteins stimulated predominantly anti-M2e IgG1 production. These results were similar with our earlier work [5] in which we studied the immunogenicity of fusion protein with one target antigen (M2e) also linked to C-terminus of FliC.

It is expected that introducing a few conserved viral epitopes into recombinant proteins for the design of universal flu vaccines should lead to enhanced protective effects [54]. The second subunit of hemagglutinin (HA2) is one such possible candidate. It is relatively conserved within viral strains from the same group and is responsible for the fusion of viral and cellular membranes in endosomes, thereby ensuring entry of the ribonucleic complexes into the cytoplasm [55]. Previously, it was shown that HA2(aa76–130)-based synthetic peptide vaccine using HA from A/Hong Kong/1/1968 (H3N2) provides protection in mice against influenza viruses of the structurally divergent subtypes H3N2, H1N1, and H5N1 [14]. Therefore, we used a conserved fragment of HA2–2(76–130) from influenza A virus group 2 as a second target antigen for design of recombinant protein with broad spectrum protection.

A number of studies of recombinant proteins have shown that the immunogenicity of an antigen depends on its location within its protein-carrier. Previous experiments of fusion proteins based on FliC have shown that the insertion of the avian influenza virus hemagglutinin globular head sequence [30, 33], the ectodomain of influenza virus M2 protein [35], as well as the F1 and V proteins of Yersinia pestis [56] into the hypervariable (immunodominant) region of flagellin were preferable for the generation of protective immune response compared with linkages onto the C- or N-terminus.

In this study, we compared the immunogenicity and protective properties of fusion proteins with a different insertion points of HA2(aa76–130) into FliC. After mouse immunization with either of the recombinant proteins, the level of antibodies capable of binding with influenza viruses of different subtypes was not very high, and the most significant anti-HA2–2 IgG response was detected against the A/H7N9 influenza virus. In the basic recombinant protein scheme used here, probably anti-HA2–2 IgG does not have a significant role in cross-protection.

Further, the results show that insertion of the HA2–2(76–130) consensus sequence into the hypervariable domain of flagellin (FlgSh-HA2–2-4M2ehs) increased M2e-specific and virus-specific CD4+ and CD8+ T-cell responses, unlike the C-terminal position of HA2–2 in the Flg-HA2–2-4M2ehs protein. Positioning of HA2–2 in the hypervariable domain of flagellin contributed to the formation of several cell types: M2e-specific CD4+ and CD8+ IFN-γ + producing; double-producing (TNF-α + IFN-γ+) T-cells; and M2e-specific CD8+ Tcm. A stronger T-cell response, however, was observed to T-cell epitopes of HA2–2. We documented a high frequency of H3N2-specific single-(IFN-γ+) and double-producing (IFN-γ + TNF-α+) CD4+, CD8+, and Tcm types in the FlgSh-HA2–2-4M2ehs group, unlike Flg-HA2–2-4M2ehs. Therefore, the insertion of HA2–2 into flagellin’s hypervariable domain induced multi-cytokine-secreting antigen-specific CD4+, CD8+, and memory T-cells. These cells have been shown to be functionally superior to single-cytokine producers in maintaining memory capacity, and they correlate with protective anti-virus immunity [57–59]. A higher frequency of multi-cytokine-secreting CD8+ T-cells was shown to be associated with decreased risk of fever, fewer symptoms, reduced illness severity score, and absence of viral shedding in individuals infected with pandemic virus. In light of those facts, it is possible that the FlgSh-HA2–2-4M2ehs fusion protein (featuring insertion of HA2–2 into the hypervariable domain) is more preferred construct in terms of developing a universal vaccine capable of delivering vigorous humoral and T-cell responses.

As expected, both proteins were able to provide protection against lethal challenge from phylogenetic group 2 influenza viruses (A/H3N2, A/H7N9). In general, there was no significant difference in protection, after lethal challenge with human (A/H3N2) and avian (A/H7N9) influenza viruses, between the two recombinant protein designs. The FlgSh-HA2–2-4M2ehs construct, however, was more effective in reducing viral load (day 6 post challenge) in the lungs of immunized mice challenged with phylogenetic group 1 (А/H5N1, А/H1N1pdm09) and 2 (A/H3N2, A/H7N9) influenza viruses compared to the Flg-HA2–2-4M2ehs construct (which showed a significant reduction only in A/H7N9 and А/H5N1).

## Conclusion

Overall, this study suggests that simultaneous expression of different M2e (M2eh and M2es) and HA2–2(76–130) sequences in recombinant protein based on flagellin induces a strong M2e-specific antibody response and HA2–2-specific CD4+ and CD8+ T-cell responses. Insertion of HA2–2(76–130) into the hypervariable domain of flagellin greatly increased antigen-specific T-cell response, as evidenced by formation of multi-cytokine-secreting T-cells and a systemic CD4+ and CD8+ response. Further studies will be directed at studying the duration of the humoral and T-cellular responses of M2e-HA2–2-Flg-based recombinant vaccine and its efficacy in protecting against phylogenetic group 1 and 2 influenza viruses.

## Additional files

Additional file 1: **Figure A, B, C.** Amino acid sequence of M2 and NA proteins of mouse-adapted A/Shanghai/2/2013(H7N9)-PR8-IDCDC. **A** Alignment of NA protein amino acid sequence of wild-type A/Shanghai/02/2013 (H7N9) virus (GenBank accession: AGL44440) and mouse adapted variant. No amino acid changes detected. **B** Alignment of HA protein amino acid sequence of wild-type A/Shanghai/02/2013 (H7N9) virus (GenBank accession: AGL44438) and mouse adapted variant. One amino acid change detected (R229I). **C** Alignment of M2 protein amino acid sequences of wild-type A/Shanghai/02/2013 (H7N9) virus (GenBank accession: AGL44442) and mouse-adapted variant. No amino acid changes detected. (PDF 294 kb)

Additional file 2: **Figure A.** Alignment of HA2 consensus sequences of A/H3N2 and A/H7N9 (including human isolates) from phylogenetic group 2. The start of HA2 subunit is indicated by the arrow; sequences of HA2(76–130) are underlined in red; identical sequences are shown in yellow; substitution by amino acids similar in properties are shown in green; amino acid substitutions are marked no color; the insertions are shown in blue. (PDF 206 kb)

Additional file 3: **Table S1.** The references relating to the B- and CD4+ T-cell epitopes in the HA2 (76–130) domain. (PDF 166 kb)

Additional file 4: **Figure A.** The gating strategy of single or double cytokine-producing antigen-specific CD4+, CD8+, Tem, and Tcm. Figure B. The dot-plots of single and double cytokine –producing M2e and virus-specific CD4 + CD44 + CD62L- in different groups. Figure C. The dot-plots of single and double cytokine –producing M2e and virus-specific CD4 + CD44 + CD62L+ in different groups. Figure D. The dot-plots of single and double cytokine –producing M2e and virus-specific CD8 + CD44 + CD62L+ in different groups. NA – non-activated cells. (PDF 2284 kb)

## Abbreviations

BSA: Bovine serum albumin
ELISA: Enzyme-linked immunosorbent assay
FBS: Fetal bovine serum
HA: Hemagglutinin
HA1: First subunit of hemagglutinin;
HA2: Second subunit of hemagglutinin
i.n.: Intranasal immunization
ICS: Intracellular cytokine staining
LD_50_: 50%-lethal dose
M2e: Ectodomain of M2 protein
MDCK: Madin-Darby canine kidney
MID: Mouse infectious dose
OD: Optical density
PBS: Phosphate buffer solution
PCR: Polymerase chain reaction
s.c.: Subcutaneous immunization
SDS-PAGE: Sodium dodecyl sulfate polyacrylamide gel electrophoresis
Tcm: Central memory T-cell
Tem: Effector memory T-cell
TLR5: Toll-like receptor 5
TMB: 3,3′, 5,5′ tetramethylbenzidine
