# Supplementary figures and images for: Flagellin-fused protein targeting M2e and HA2 induces potent humoral and T-cell responses and protects mice against various influenza viruses a subtypes
Source: J Biomed Sci. 2018 Apr 9;25:33. doi: 10.1186/s12929-018-0433-5 (PMC5891888; doi:10.1186/s12929-018-0433-5)

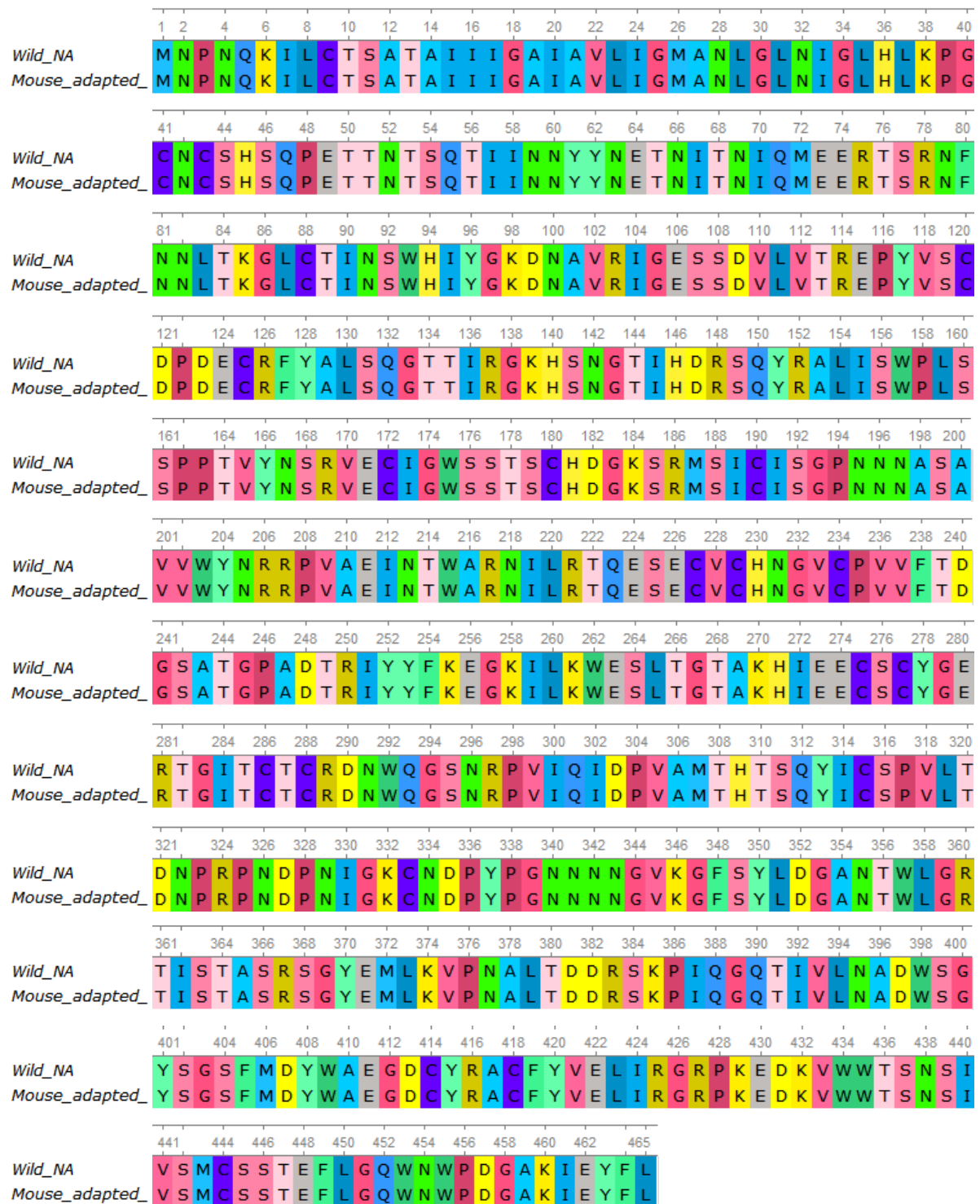

**Figure A**

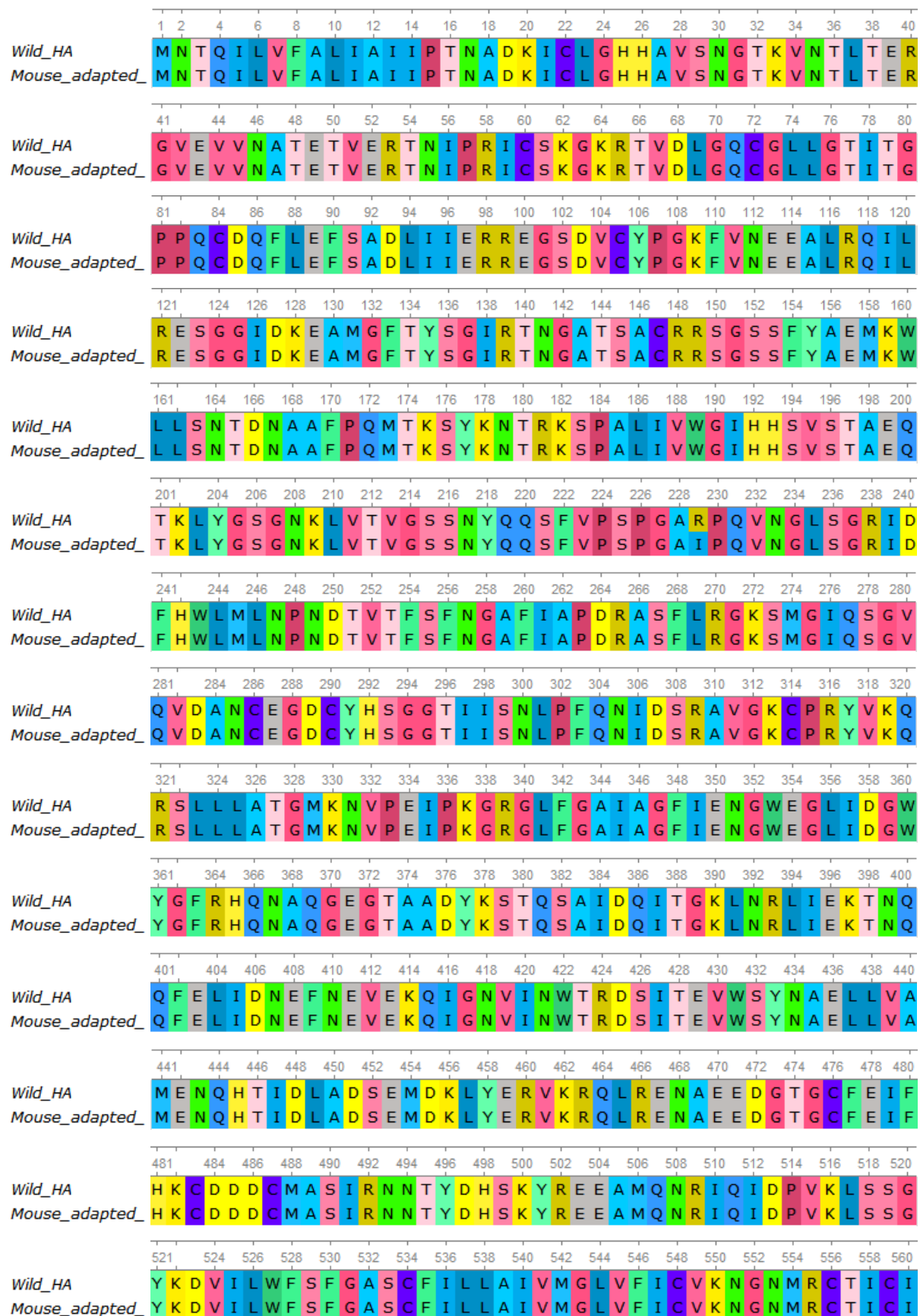

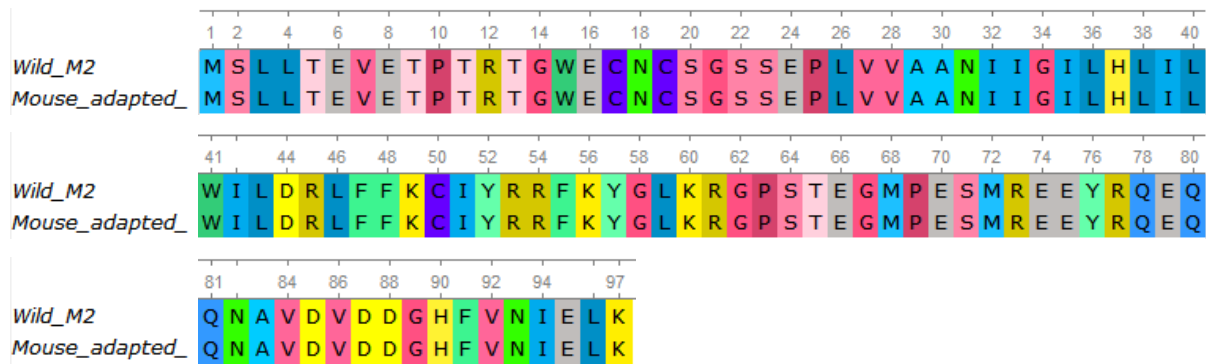

**Figure C**

Supplement: Supplementary file 1 — Figure A, B, C. Amino acid sequence of M2 and NA proteins of mouse-adapted A/Shanghai/2/2013(H7N9)-PR8-IDCDC. A Alignment of NA protein amino acid sequence of wild-type A/Shanghai/02/2013 (H7N9) virus (GenBank accession: AGL44440) and mouse adapted variant. No amino acid changes detected. B Alignment of HA protein amino acid sequence of wild-type A/Shanghai/02/2013 (H7N9) virus (GenBank accession: AGL44438) and mouse adapted variant. One amino acid change detected (R229I). C Alignment of M2 protein amino acid sequences of wild-type A/Shanghai/02/2013 (H7N9) virus (GenBank accession: AGL44442) and mouse-adapted variant. No amino acid changes detected. (PDF 294 kb) [file 12929_2018_433_MOESM1_ESM.pdf]

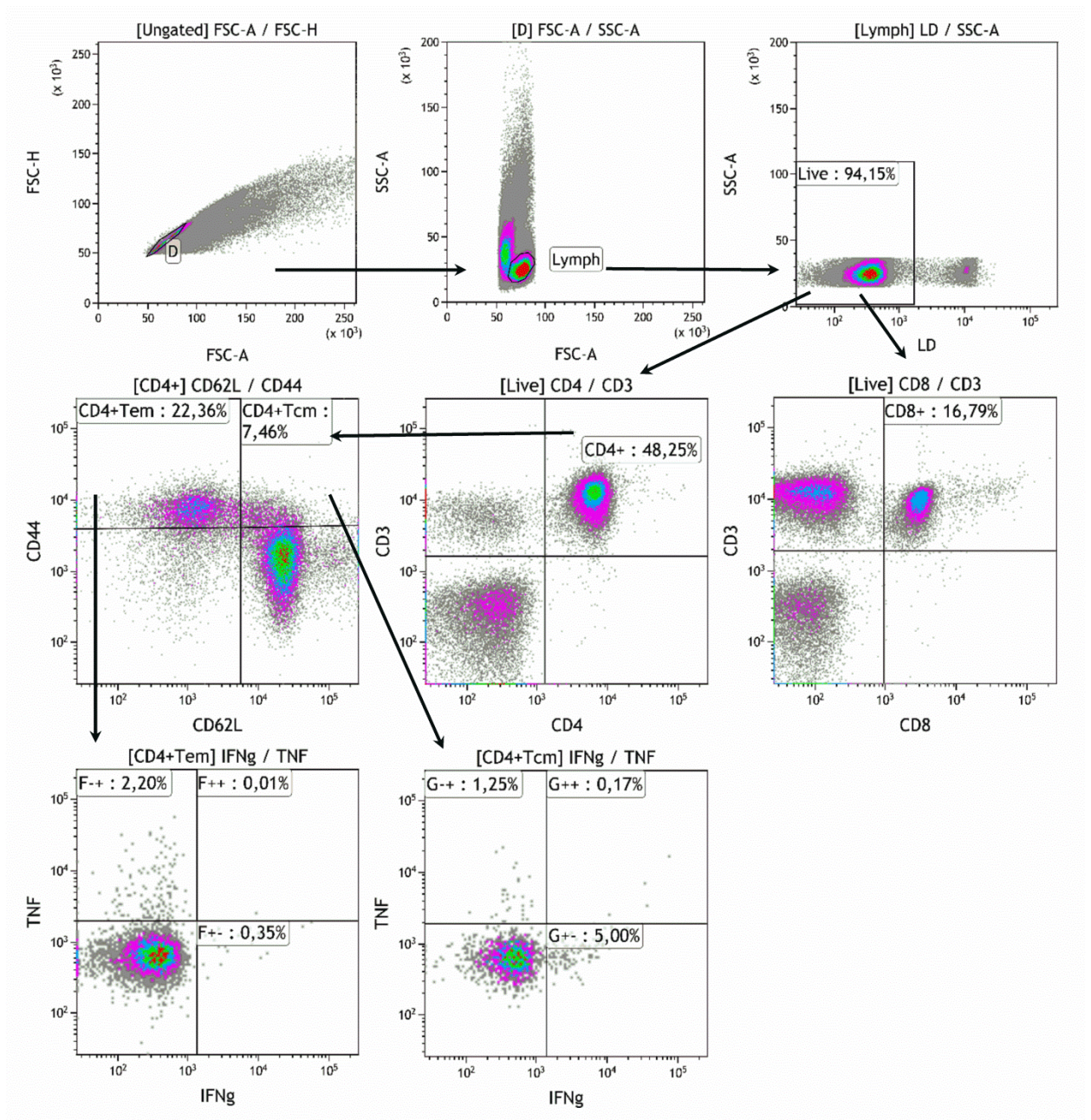

**Figure A**

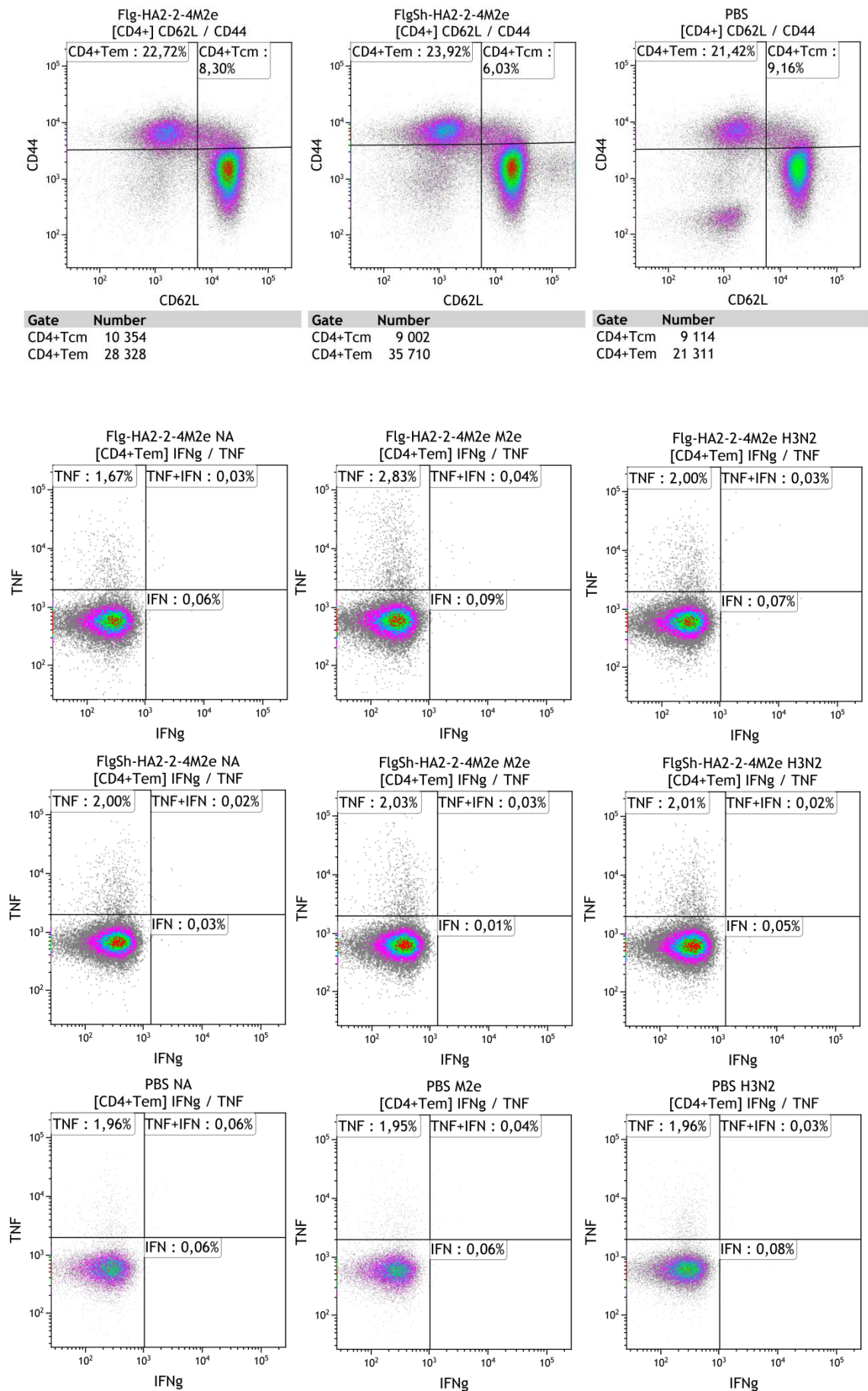

**Figure B**

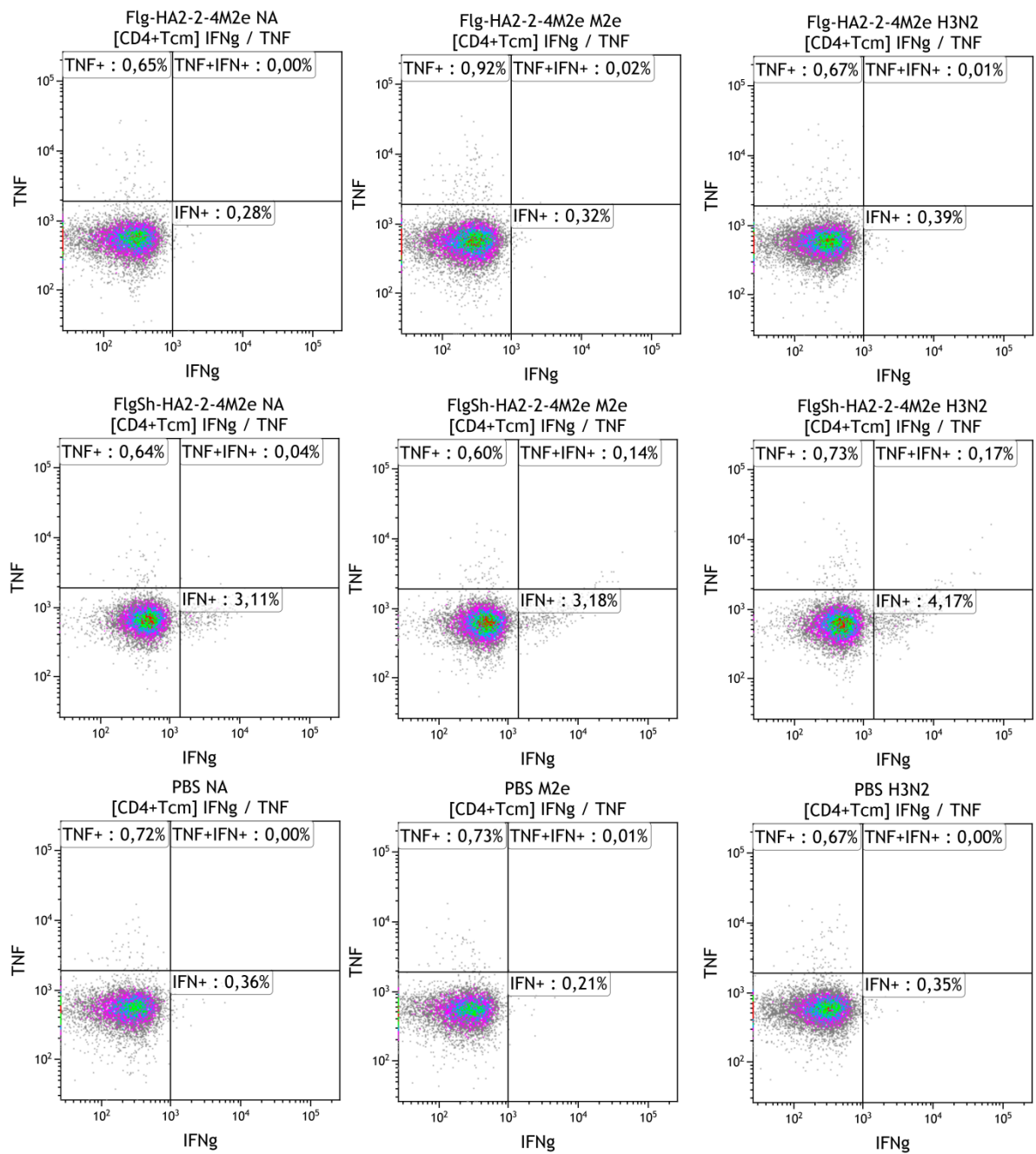

**Figure C**

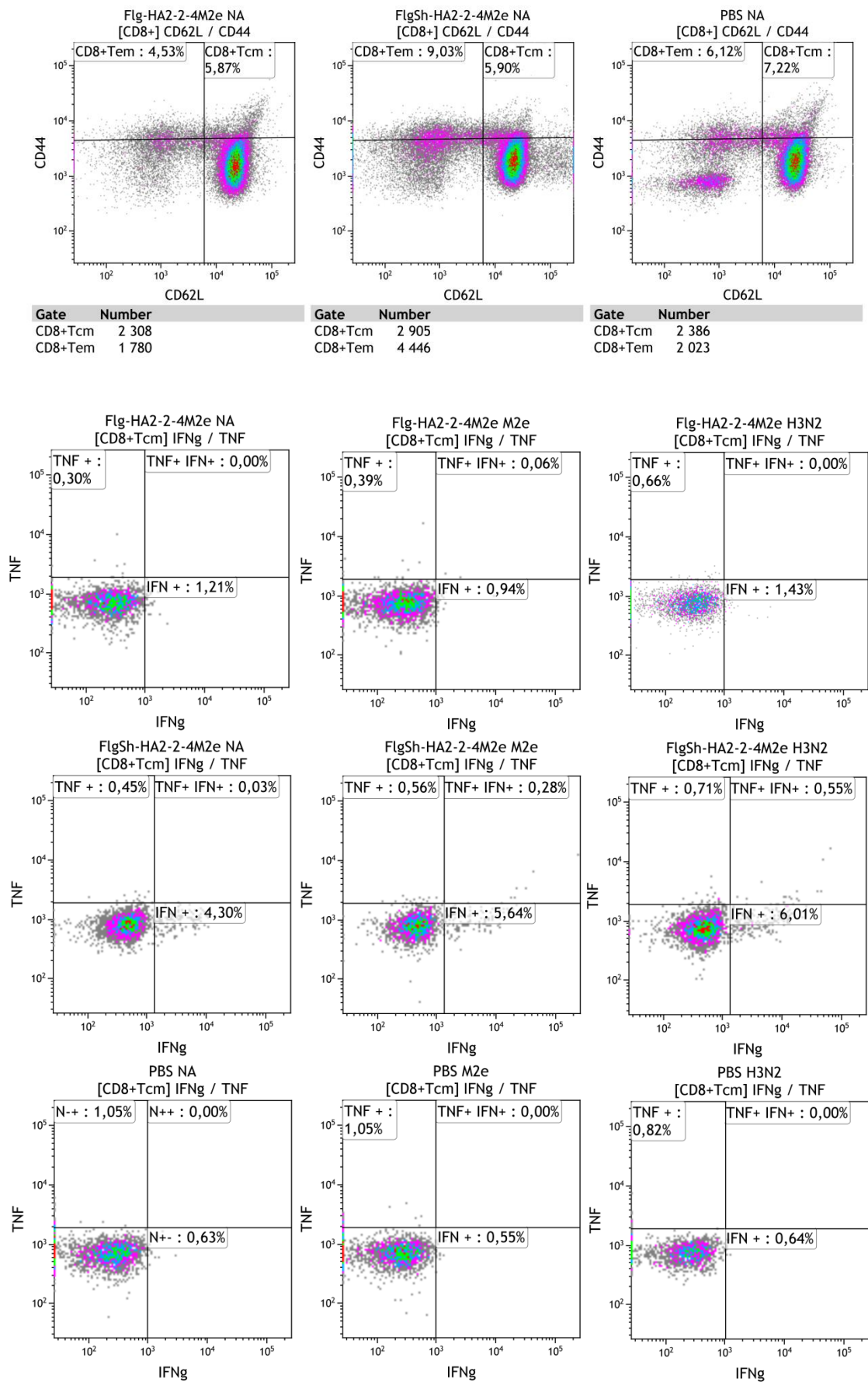

**Figure D**

Supplement: Supplementary file 4 — Figure A. The gating strategy of single or double cytokine-producing antigen-specific CD4+, CD8+, Tem, and Tcm. Figure B. The dot-plots of single and double cytokine –producing M2e and virus-specific CD4 + CD44 + CD62L- in different groups. Figure C. The dot-plots of single and double cytokine –producing M2e and virus-specific CD4 + CD44 + CD62L+ in different groups. Figure D. The dot-plots of single and double cytokine –producing M2e and virus-specific CD8 + CD44 + CD62L+ in different groups. NA – non-activated cells. (PDF 2284 kb) [file 12929_2018_433_MOESM4_ESM.pdf]
