# Supplementary material for: Flagellin-fused protein targeting M2e and HA2 induces potent humoral and T-cell responses and protects mice against various influenza viruses a subtypes
Source: J Biomed Sci. 2018 Apr 9;25:33. doi: 10.1186/s12929-018-0433-5 (PMC5891888; doi:10.1186/s12929-018-0433-5)
Supplement: Supplementary file 3 — Table S1. The references relating to the B- and CD4+ T-cell epitopes in the HA2 (76–130) domain. (PDF 166 kb) [file 12929_2018_433_MOESM3_ESM.pdf]

Table 1

Epitope ID	Epitope	References
13575	ENQHTIDLTDSEMNKLFEKTRKQLRENAEDMGNGCF	Gelder CM et al. 1995, J Virol
22098	GRIQDLEKYVEDTKIDLWS	Gelder CM et al. 1995, J Virol
30386	KEFSEVEGRIQDLEKYV	Simeckova-Rosenberg J et al. 1995, Vaccine
		Atassi MZ et al. 1984, Immunol Commun
31200	KIDLWSYNAELLVALE	Gelder CM et al. 1998, Int Immunol
31201	KIDLWSYNAELLVALENQHTI	Gelder CM et al. 1995, J Virol
36498	LIEKTNEKFHQIEKEFSEVEGRIQDLEKYVEDTKI	Gelder CM et al. 1995, J Virol
50488	QDLEKYVEDTKIDLWS	Gelder CM et al. 1995, J Virol
50489	QDLEKYVEDTKIDLWSYNAELLVALENQHTIDLTDS	Gelder CM et al. 1995, J Virol
62654	SYNAELLVALENQHTI	Gelder CM et al. 1995, J Virol
97636	SEVEGRIQDLEKYVEDTK	Laurel Yong-Hwa Lee et al. 2008, J Clin Invest
129078	KIDLWSYNAELLVALEN	Jenny Aurielle B Babon et al. 2009, Hum Immunol
129760	RIQDLEKYVEDTKIDLW	Jenny Aurielle B Babon et al. 2009, Hum Immunol
130384	YNAELLVALENQHTIDL	Jenny Aurielle B Babon et al. 2009, Hum Immunol
422849	WSYNAELLVAMENQHTI	Katherine A Richards et al. 2015, J Infect Dis
		Anthony DiPiazza et al. 2017, Clin Vaccine Immunol
