# Supplementary material for: Flagellin-fused protein targeting M2e and HA2 induces potent humoral and T-cell responses and protects mice against various influenza viruses a subtypes
Source: J Biomed Sci. 2018 Apr 9;25:33. doi: 10.1186/s12929-018-0433-5 (PMC5891888; doi:10.1186/s12929-018-0433-5)
Supplement: Supplementary file 2 — Figure A. Alignment of HA2 consensus sequences of A/H3N2 and A/H7N9 (including human isolates) from phylogenetic group 2. The start of HA2 subunit is indicated by the arrow; sequences of HA2(76–130) are underlined in red; identical sequences are shown in yellow; substitution by amino acids similar in properties are shown in green; amino acid substitutions are marked no color; the insertions are shown in blue. (PDF 206 kb) [file 12929_2018_433_MOESM2_ESM.pdf]

```
                           1                                                50
Consensus H3      (1)  MKTIIALSYILCLVFAQKLPXGNDNSTATLCLGHHAVPNGTLVKTITNDQ
Consensus H7      (1)  -----MNTQILVFALCAIIP----TNADKICLGHHAVSNGTKVNTLTERG

                       51                                              100
Consensus H3     (51)  IEVTNATELVQSSSTGRICDSPHQILDGENCTLIDALLGDPHCDGFQNKE
Consensus H7     (42)  VEVVNATETVERTNIPRICSKGKRTVDLGQCGLLGTITGPPQCDQFLEFS
                       101                                             150
Consensus H3    (101)  WDLFVERSKAYSNCYPYDVPDYASLRSLVASSGTLEFNNESFNWTGVAQN
Consensus H7     (92)  ADLIIERREGSDVCYPGKFVNEEALRQILRESGGIDKESMGFTYSGIRTN
                       151                                             200
Consensus H3    (151)  GTSSACKRRSVKSFFSRLNWLHX--LKYKYPALNVTMPNNEKFDKLYIWG
Consensus H7    (142)  GATSACRR-SGSSFYAEMKWLLSNTDNAAFPQMTKSYKNTRKKPALIIWG
                       201                                             250
Consensus H3    (199)  VHHPSTDSDQISLYAQASGRVTVSTKRSQQTVIPNIGSRPWVRGVSSRIS
Consensus H7    (191)  IHHSGSTTEQTKLYGSGNKLITVGSSNYQQSFVPSPGARPQVNGQSGRID
                       251                                             300
Consensus H3    (249)  IYWTIVKPGDILLINSTGNLIAPRGYFKIRSGKSSIMRSDAPIGKCNSEC
Consensus H7    (241)  FHWLMLNPNDTVTFSFNGAFIAPDRASFLRGKSMGIQSGVQVDANCEGDC
                       301                                             350
Consensus H3    (299)  ITPNGSIPNDKPFQNVNRITYGACPRYVKQNTLKLATGMRNVPEKQ----
Consensus H7    (291)  YHSGGTIISNLPFQNINSRAVGKCPRYVKQESLLLATGMKNVPEIPKGSR
                       351↓                                             400
Consensus H3    (345)  -TRGIFGAIAGFIENGWEGMVDGWYGFRHQNSEGTGQAADLKSTQAAIXQ
Consensus H7    (341)  VGRGLFGAIAGFIENGWEGLIDGWYGFRHQNAQGEGTAADYKSTQSAIDQ
                       401                                             450
Consensus H3    (394)  INGKLNRLIEKTNEKFHQIEKEFSEVEGRIQDLEKYVEDTKIDLWSYNAE
Consensus H7    (391)  ITGKLNRLIEKTNQQFELIDNEFNEVEKQIGNVINWTRDSMTEVWSYNAE
                       451                                             500
Consensus H3    (444)  LLVALENQHTIDLTDSEMNKLFERTRKQLRENAEDMGNGCFKIYHKCDNA
Consensus H7    (441)  LLVAMENQHTIDLADSEMNKLYERVKRQLRENAEEDGTGCFEIFHKCDDD
                       501                                             550
Consensus H3    (494)  CIGSIRNGTYDHDVYRDEALNNRFQIKGVELKSGYKDWILWISFAISCFL
Consensus H7    (491)  CMASIRNNTYDHSKYREEAMQNRIQIDPVKLSSGYKDVILWFSFGASCFI
                       551                    574
Consensus H3    (544)  LCVVLLGFIMWACQKGNIRCNICI
Consensus H7    (541)  LLAIAMGLVFICVKNGNMRCTICI
```

Figure A
